# Correlation of online assessment parameters with summative exam performance in undergraduate medical education of pharmacology: a prospective cohort study

**DOI:** 10.1186/s12909-019-1814-5

**Published:** 2019-11-08

**Authors:** Felizian Kühbeck, Pascal O. Berberat, Stefan Engelhardt, Antonio Sarikas

**Affiliations:** 1 Technical University of Munich, Institute of Pharmacology and Toxicology, Biedersteiner Str. 29, 80802 Munich, Germany; 2 Technical University of Munich, School of Medicine, Center of Medical Education, Ismaninger Str. 22, 81675 Munich, Germany; 3 Paracelsus Medical University, Institute of Pharmacology and Toxicology, Strubergasse 21, 5020 Salzburg, Austria

**Keywords:** Computer-assisted assessment, Online assessment, Written assessment, Prediction, Gender differences, Summative assessments, Undergraduate education, Pharmacology

## Abstract

**Background:**

Learning analytics aims to improve learning outcomes through the systematic measurement and analysis of learning-related data. However, which parameters have the highest predictive power for academic performance remains to be elucidated. The aim of this study was to investigate the correlation of different online assessment parameters with summative exam performance in undergraduate medical education of pharmacology.

**Methods:**

A prospective study was conducted with a cohort of undergraduate medical students enrolled in a pharmacology course at Technical University of Munich, Germany. After a four-week teaching and learning period, students were given access to an online assessment platform consisting of 440 multiple choice (MC) questions. After 12 days, a final written summative exam was performed. Bivariate correlation and multiple regression analyses were performed for different online assessment parameters as predictors and summative exam performance as dependent variable. Self-perceived pharmacology competence was measured by questionnaires pre- and postintervention.

**Results:**

A total of 224 out of 393 (57%) students participated in the study and were included in the analysis. There was no significant correlation for the parameters “number of logins” (*r* = 0.01, *p* = 0.893), “number of MC-questions answered” (*r* = 0.02, *p* = 0.813) and “time spent on the assessment platform” (*r* = − 0.05, *p* = 0.459) with exam performance. The variable “time per question” was statistically significant (*p* = 0.006), but correlated negatively (*r* = − 0.18) with academic performance of study participants. Only “total score” (*r* = 0.71, *p* < 0.001) and the “score of first attempt” (*r* = 0.72, *p* < 0.001) were significantly correlated with final grades. In a multiple regression analysis, “score first attempt” accounted for 52% of the variation of “score final exam”, and “time per question” and “total score” for additional 5 and 1.4%, respectively. No gender-specific differences were observed. Finally, online assessments resulted in improved self-perceived pharmacology competence of students.

**Conclusion:**

In this prospective cohort study, we systematically assessed the correlation of different online assessments parameters with exam performance and their gender-neutrality. Our findings may help to improve predictive models of academic performance in undergraduate medical education of pharmacology.

## Background

In Germany, undergraduate medical education of pharmacology is typically subdivided into general pharmacology, taught in the first clinical year, and clinical pharmacology / pharmacotherapy, presented in the third clinical year [1]. Despite substantial efforts to improve pharmacology training at German medical schools, a survey by Ochsmann et al. [2] showed that the majority of graduates feel ill-prepared for drug therapy-related problems. To address this problem, we sougth to develop an online assessment platform to monitor pharmacology knowledge of undergraduate medical students by learning analytics. During the last decade, learning analytics has emerged as a significant area of research into technology-enhanced learning [3], prompted by the emergence of online learning and the ubiquity of the internet in higher education [4]. As set out by the first International Conference on Learning Analytics and Knowledge (LAK 2011) and adopted by the Society for Learning Analytics Research (SoLAR), learning analytics is defined as “the measurement, collection, analysis and reporting of data about learners and their context, for purposes of understanding and optimizing learning and the environments in which it occurs”. In theory, learning analytics may provide valuable feedback to both learners and teachers, leading to a “personalization” of the teaching and learning process with added efficacy [5]. Moreover, predicting performance via learning analytics tools may be beneficial for monitoring students who are at risk for removal from the register of students due to repeated failing of exams.

However, learning analytics is still in the early stages of implementation [6]. At many educational institutions, learning analytics is based on track data from learning management systems (LMS) that log learner activities, e.g. the number of clicks, the time spent in LMS, or the participation in online discussion forums. Other systems include data from computer-assisted assessments or data retrieved from students’ admission systems, such as accounts of previous education [7]. In addition, there is considerable controversy surrounding the question what learning analytics data are most suited to model learning processes or to predict academic performance [7, 8]. Furthermore, it has been asserted that the use of computers in higher education [12–14] and learning outcomes in online courses [15–17] are increasingly gender neutral. However, in the area of computer-assisted assessments, research is not conclusive. Finally, enabling students to make accurate judgements about their performance is an implicit aim of higher education [9, 10]. However, it was shown that the usual self-assessment has only a poor accuracy when compared to performance [11].

In the present manuscript, we conducted a prospective study to systematically investigate the following research questions with a cohort of undergraduate medical students in pharmacology: i) Which online assessment parameters have the highest correlation with summative exam performance? ii) Do online assessments help students to better judge their self-perceived pharmacology competence?, and iii) is there a gender difference regarding online assessments?

## Methods

### Study design and participants

**Fig. 1:**
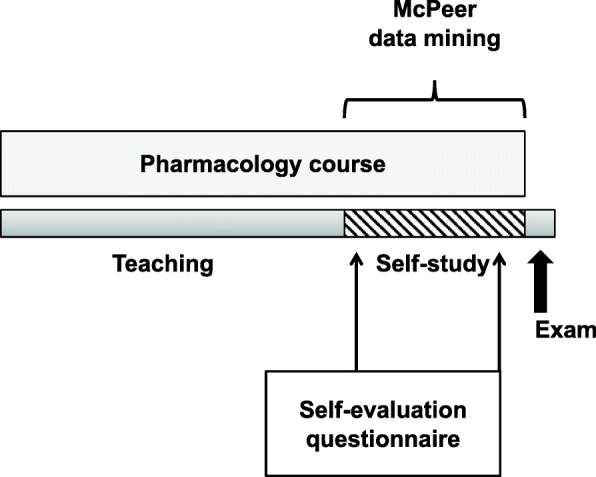
Experimental setting and timeline. A mixed-methods design of quantitative studies (McPeer data mining and self-evaluation questionnaires) was employed during an undergraduate pharmacology course at Technical University of Munich, Germany. The course consisted of a 24-day teaching period with daily lectures and twice-weekly seminars, followed by a 12-day self-study period and a final written exam

A prospective explanatory design was employed to investigate the potential of different online assessments parameter for the prediction of student performance in undergraduate medical education of pharmacology. The study was conducted with a cohort of 393 first-year medical students enrolled in a general pharmacology course at Technical University of Munich (TUM). The module consisted of a 24-day teaching and learning period with daily lectures on weekdays and twice-weekly seminars, followed by a 12-day self-study period and a final written exam (Fig. 1).

Quantitative data were collected during the self-study period by an online assessment platform (designated McPeer, available at www.mcpeer.de) that was custom-programmed for the purpose of this study (Additional file 1: Figure S1). This approach was chosen to limit confounding variables (e.g. a differing format, style or thematic focus of test questions when compared to the teaching module), and to further develop and optimize the platform based on the results of this study. The assessment platform was made available to the study participants after the teaching period to ensure a uniform baseline pharmacology knowledge and to limit its use as primary learning source. Online self-evaluation questionnaires were displayed after the first login to McPeer (1st rating, pre-intervention) and 24 h before the final exam (2nd rating, post-intervention) to obtain data on self-perceived pharmacology competence. The study protocol and consent procedure were approved by the ethics committee of the TUM School of Medicine (project number 564/15 S). Study participation was voluntary, and informed consent was obtained from all study participants via an online form. All data were processed in a pseudonymized manner.

### Data collection and instruments

To collect quantitative data in real time, we developed a web-based learning analytics platform. The platform was written in Hypertext Preprocessor (PHP) as server-side programming language to be compatible with all main operating systems and linked to a My Structured Query Language (MySQL) database. The learning analytics platform was accessible via the internet at http://www.mcpeer.de. The database contained 440 multiple-choice (MC) questions of the single-best answer type with five alternate answers (Additional file 1: Figure S1C). The questions were divided into 27 sets that covered all relevant course topics (Additional file 6: Table S1). The average number of MC-questions per set was 16.3 ± 6.1 with a mean of 35.2 ± 17.3 words per question. All MC-questions were authored by pharmacology lecturers at TUM and checked for equal discriminatory power and difficulty.

After log on, study participants could oversee their progress for each topic (total number of questions, number of answered questions and percentage correctly answered questions) and start question sessions (Additional file 1: Figure S1B). Results were displayed instantly by highlighting the correct answer (Additional file 1: Figure S1C). There was no time limit or restriction on the number of repetitions for each question set. The following learning analytics parameters were automatically logged by the assessment platform: number of logins, total questions answered, total score, score of each attempt, total time spent on the platform and time required for answering a question. In addition, a subgroup analysis was conducted for study participants who completed two full rounds of question sets, thus solving all MC-questions at least twice.

The final exam was a paper-based summative assessment that consisted of 50 MC-questions of the single-best answer type with five answer options. The questions were newly written by TUM pharmacology lecturers, have not been presented to the study cohort before and covered all relevant topics of the McPeer assessment platform. The online self-evaluation questionnaire solicited the self-perceived pharmacology competency on a 5-point Likert scale (1 = “not confident” to 5 = “confident”) for each of the 27 course topics (Additional file 2: Figure S2 and Additional file 6: Table S1). In this context, “confident” was defined as a “sound understanding of basic concepts of pharmacology (e.g. pharmakokinetics and mechanisms of action of representative drugs), while “not confident” was defined as an inadequate knowledge of basic drugs and mechanisms. Participants could opt not to answer each item. Validity evidence for all questionnaires was collected using a two-step process. First, content validity was established through evaluation of the questionnaires by fellow lecturers at TUM. Second, questionnaires were pilot tested with a subset of undergraduate medical students.

### Pseudonymization of data

Unique identifier codes were generated for each study participant to match individual student data with assessment results in a pseudonymized manner. The lecturers of the pharmacology course had no access to research data.

### Statistics

To determine the variation of different assessment parameters and exam performance, data was analyzed by Pearson’s correlation coefficient (*r*). A multiple regression model with a forward variable selection algorithm was applied to take all other variables into account and to estimate how much explanatory value each variable provide by using r-square and r-square changes. Model assumptions were tested by performing a residual analysis. Normality of distributions was tested with the Kolmogorov-Smirnov test. In addition, skewness and kurtosis was calculated for the analyzed variables. Wilcoxon signed-rank test was used to analyze differences between pairs of observations collected during the self-assessment. Continuous variables were described by using the mean and standard deviation. Groups were compared by Mann-Whitney U test or Student’s t-test. For the analysis of the difference between two dependent correlations from the same sample, Steiger’s z-test was used. Fisher’s r-to-z transformation was applied on the correlation coefficients to obtain z-scores which can be compared in an asymptotic z-test. Significance is considered for z-scores greater than |1.96| for a two-tailed test. *p*-values < 0.050 were considered statistically significant. All statistical calculations were performed with the Statistical Package for the Social Sciences (SPSS), version 23 (IBM Corporation, Armonk, NY). Histograms and box plots were constructed with GraphPad PRISM 6.0 software (La Jolla, CA).

## Results

### Demographic data

**Fig. 2:**
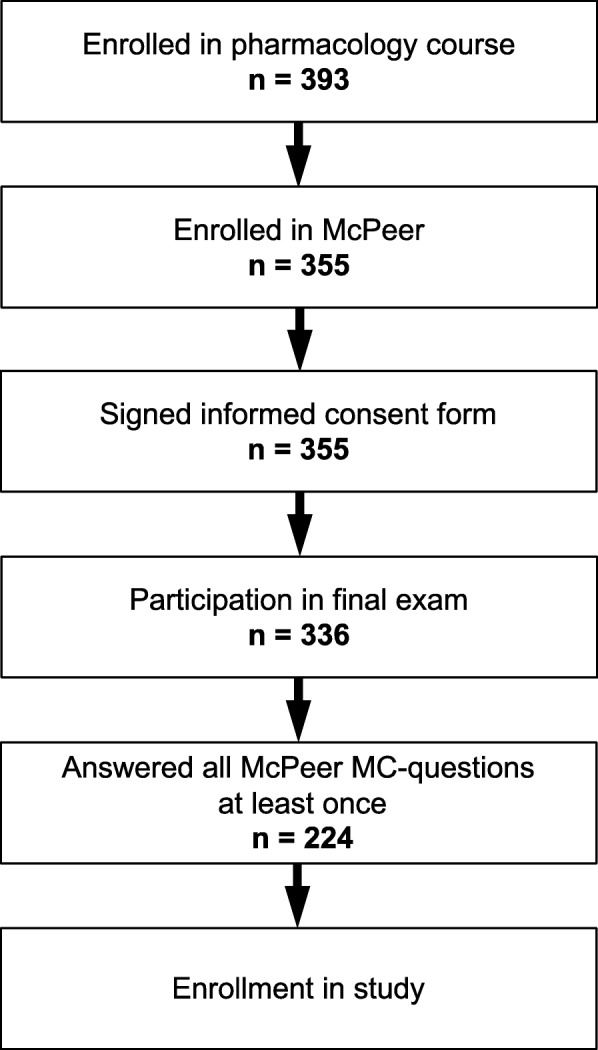
Flow chart of study design. Of 393 first-year medical students enrolled in a general pharmacology course at Technical University of Munich, Germany, 224 (57%) participated in the study

A total of 224 out of 393 (57%) students enrolled in the pharmacology course (winter term 2014/15) at TUM participated in the study (Fig. 2). The mean age of participants was 24.4 ± 4.2 years. The female:male ratio of participants was 150:74 with 150 (67%) female and 74 (33%) male. In comparison, 65% of all medical students and 67.7% of first-year medical students in Germany in the winter term 2014/15 were female [18].

### Correlations of online assessment variables with final grade

To identify potential correlational trends between online assessment variables and final grade as a measure of academic performance, we developed scatter plots as initial approach as described previously [19, 20]. Additional file 3: Figure S3 depicts representative scatter plots of selected variables versus student final grade. The mean percentage of correct answers in the final exam was 73.6 ± 12.8% with females 73.8 ± 13.2% and males 73.2 ± 12.0%.

To further interrogate the significance of selected variables as indicators of student achievement, a simple (bivariate) correlation of each variable with student final grade was performed. Of the objective variables logged by the assessment platform, only total score and score of the first attempt had a positive and statistically significant correlation with student final grade (*r* = 0.71 and *r* = 0.72, respectively, *p* < 0.001) (Table 1). In contrast, there was no significant correlation between the number of logins (*r* = 0.01, *p* = 0.893), number of MC-questions answered (*r* = 0.02, *p* = 0.813) or time spent on the assessment platform (*r* = − 0.05, *p* = 0.459) with final grades. The variable “time per question” was statistically significant (*p* = 0.006), but correlated negatively (*r* = − 0.18) with academic performance of study participants. In summary, bivariate correlation analysis yielded total score and score of the first attempt as objective, loggable variables with a positive and statistically significant correlation with final grades as a surrogate of academic performance.

**Table 1:** Descriptive statistics and bivariate correlation of different online assessment parameters with summative exam results

Parameter	Mean (± SE)	Median	Bivariate correlation *r*	*p*-value
Number of logins	10.01 (± 7.01)	9 [5;14]	0.01	0.893
Total questions	813.82 (± 378.41)	701 [532;945]	0.02	0.813
Total score	75.45% (± 9.18)	76 [69;82]	0.71	< 0.001
Score first attempt	70.24% (± 10.14)	71 [64;77]	0.72	< 0.001
Total time	4.99 h (± 1.83)	5 [4;6]	- 0.05	0.465
Time per question	25.71 s (± 1.73)	26 [25;27]	- 0.18	0.006

We next performed a subgroup analysis to study the effect of repeated administration of assessment items on Pearson’s correlation coefficient *r*. A common problem of repeated measure designs is the possibility of serial order carryover effects [21] that lead to testing artifacts. These may result in performance improvements (e.g. by learning effects) or declines (e.g. by decreased motivation) in assessments. Interestingly, our study participants performed significantly better in the second attempt when compared to the first administration of questions (68.09 ± 12.13% vs. 81.83 ± 10.20% correct answers, *p* < 0.001; *n* = 46) (Additional file 4: Figure S4). However, bivariate correlations of online formative test scores with final grade at first administration (*r* = 0.8, *p* < 0.001) and second administration (*r* = 0.75, *p* < 0.001) were in a similar range and did not differ with statistical significance (z-score = 1.08, *p* = 0.275).

Next, we performed a multiple regression analysis for the following variables: “number of logins”, “total questions”, “total score”, “score first attempt”, “total time”, “time per question” as predictors and “score final exam” as the dependent variable. A stepwise forward variable selection algorithm was applied and “number of logins”, “total questions” and “total time” was removed from the final model, while “total score”, “score first attempt” and “time per question” were included in the final model (Table 2). The best univariate predictor (“score first attempt”) achieves an *r*^2^ = 0.52, the multiple regression model achieves *r*^2^ = 0.60 (adjusted *r*^2^ = 0.59) suggesting that the multivariate approach explains additional 8% of the variation of “score final exam”. In the multiple regression and after controlling for all the other variables, “score first attempt” accounts for 52% of the variation of “score final exam”, “time per question” for additional 5% and “total score” for additional 1.4%. In summary, the results of the multiple regression analysis confirmed our univariate results, demonstrating that the suggested predictors are useful, even when used in a multivariate approach.

**Table 2:** Multiple regression analysis. A stepwise forward variable selection algorithm was applied and “number of logins”, “total questions” and “total time” was removed from the final model. The parameters “total score”, “score first attempt” and “time per question” were included in the final model

Variable	Effect change	SE	t-value	r-square	*p*-value
Score first attempt	0.67	0.11	6.03	0.52	< 0.0000001
Time per question	−1.56	0.33	−4.75	0.05	0.000004
Total score	0.34	0.12	2.73	0.014	0.007

Collectively, these results show that of all objective variables logged by the online assessment platform, the cumulative score of MC-questions has the highest correlation to summative exam results. In addition, our data indicate that already the result of the first attempt is a valid predictor of academic performance.

### Self-assessment of knowledge is a weak predictor of academic performance

**Fig. 3:**
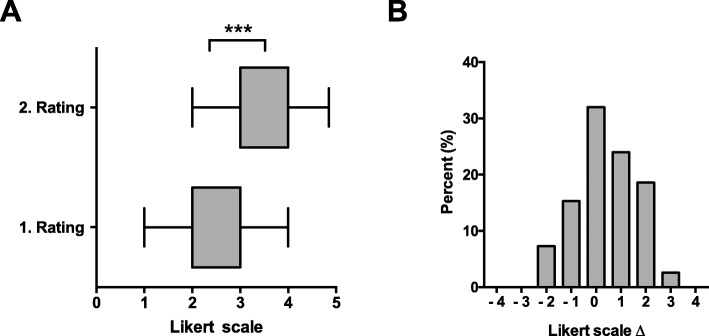
Pre- and post-intervention assessment of self-perceived pharmacology competency. Online questionnaires were displayed at first login to McPeer (1. rating, preintervention) and 24 h before the final exam. A 5-point Likert-scale (1 = “insecure” to 5 = “secure”) was used. **a** Box plots showing mean, first and third quartile with whiskers representing the 5 and 95% percentile, *n* = 224. *** = *P* < 0.001 (t-test). **b** Differences between pairs of self-assessments (Likert **Δ**) as calculated by sign-tests before and after use of McPeer. *n* = 224

Next, we studied the potential of subjective variables by the students as predictors of academic performance using their final grade as comparison. As the capacity of students to make judgements about their work is an implicit aim of higher education [9, 10], we chose self-assessment, the process by which a learner judges the quality and quantity of his/her learning [22] as an subjective parameter for further investigation. For this purpose, study participants were presented an online questionnaire at first login to the online assessment platform (1st rating, pre-intervention) and 24 h before the final exam (2nd rating, post-intervention) (Fig. 1). A total of 157 out of 224 students (70%) students submitted both questionnaires. The mean score of the post-intervention rating was markedly increased when compared to the pre-intervention score (mean Likert scale rating 3.39 ± 0.82 vs. 2.67 ± 0.89, median 3 [2;3] vs. 3 [3;4]) (Fig. 3a). Of note, bivariate correlation of the 1st and 2nd rating with final exam grades yielded a Pearson’s correlation coefficient of *r* = 0.28 (*p* < 0.001) and *r* = 0.46 (*p* < 0.001), respectively, that was statistically significant (z-score = − 2.23; *p* = 0.025). For a more in-depth analysis of differences between pre- and postintervention self-assessment scores, Wilcoxon signed-rank tests were performed (Fig. 3b). These revealed significant changes between pre- and postintervention Likert ratings (*p* < 0.001). Of 157 study participants, 55% (*n* = 86) had a higher Likert score in the postintervention rating, suggesting an improved self-perceived pharmacology competency upon completion of the online assessments. In contrast, 4% (*n* = 7) showed a lower Likert score on the postintervention questionnaire and 41% (*n* = 64) of participants were found to have unchanged Likert scores. Collectively, these results suggest that self-assessment of knowledge is a weak but valuable variable for performance prediction. In addition, these results support the notion that online assessments may improve and “calibrate” the predictive accuracy of knowledge self-assessments.

### Gender-specific analysis of prediction variables

To address the question of gender-specific differences with respect to computer-assisted assessments, we systematically performed bivariate testing for both gender groups (Additional file 7: Table S2). Similar to the results for the whole cohort, bivariate correlation of variables to final exam score were positive and statistically significant only for total score (male: *r* = 0.71, *p* < 0.001; female: *r* = 0.72, *p* < 0.001) and score of the first attempt (male: *r* = 0.77, *p* < 0.001; female: *r* = 0.71, *p* < 0.001) for male and female students. The variable “time per question” showed a weak correlation (male: *r* = − 0.25; female: *r* = 0.16), but was statistically significant only for male study participants (*p* = 0.034). Two-tailed t-tests revealed no significant differences between male and female participants for all variables studied (Additional file 7: Table S2). Interestingly, we observed a statistically significant difference between pre- and postintervention self-assessments, in which male study participants (1st rating: 3.0 ± 0.97; 2nd rating: 3.6 ± 0.91) judged their pharmacology competency significantly (Mann-Whitney U test) higher (*p* < 0.001) than female students (1st rating: 2.5 ± 0.81; 2nd rating: 3.3 ± 0.76) (Additional file 5: Figure S5). However, no significant differences were observed when correlating gender-specific results of self-assessment scores with final exam grades (1st rating: male *r* = 0.29 vs *r* = female 0.29, 2nd rating: male *r* = 0.46 vs female *r* = 0.47). In summary, these results confirmed total score and the score of the first attempt as gender-neutral parameters with the highest correlation with exam performance.

## Discussion

In this prospective study, we systematically investigated the correlation of different online assessment parameters with summative exam performance in undergraduate medical education of pharmacology. Our results revealed no significant correlation of the variables “number of logins”, “number of MC-questions answered” or “time spent on the assessment platform” with final grades. The variable “time per question” was statistically significant, but correlated negatively with academic performance of study participants. Only “total score” and the “score of first attempt” were significantly correlated with exam performance. In a multiple regression analysis, “score first attempt” accounted for 52% of the variation of “score final exam”, and “time per question” and “total score” for additional 5 and 1.4%, respectively. In addition, analysis of self-evaluation questionnaires indicated that online assessments resulted in improved self-perceived pharmacology competence of students. Finally, this study found no gender-specific differences in predictive modeling of academic performance by online assessments. Collectively, the results of this study may help to improve predictive models of academic performance in undergraduate medical education of pharmacology.

### Positive correlation of online assessment scores with exam performance

In our study, we found that the best univariate predictor (“score first attempt”) had an *r*^2^ = 0.52 and *r*^2^ = 0.60 (adjusted *r*^2^ = 0.59) in the multiple regression model, indicating that the multivariate approach explains an additional 8% of the variation of the parameter “score final exam”. In the multiple regression and after controlling for all the other variables, “score first attempt” accounts for 52% of the variation of “score final exam”, “time per question” for additional 5% and “total score” for additional 1.4%. The results of this study conducted with undergraduate medical students in Germany substantiate previous findings in literature that online formative assessments positively correlate with exam achievements and may be useful for predictive modeling of student performance. Tempelaar and co-workers showed that longitudinal computer-assisted formative assessments in a mathematics and statistics course at the Business & Economics school at Maastricht University are the best predictor for detecting underperforming students and academic performance [8]. The authors concluded that “true assessment data”, even if these come from assessments that are more of the formative that of the summative type, are the most reliable predictor. This concurs well with Wolff et al. who showed that performance on initial assessments during the first parts of online modules were substantial predictors for final exam performance [23]. Our study extends these findings by the observation that already results of the first attempt when answering MC-question based online assessments have a high predictive potential that does not statistically differ from repeated test results.

### Student activity data has a poor correlation with academic performance

In contrast, we found that the variables “number of logins”, “total questions answered” and “time spent on the assessment platform” do not significantly correlate with exam performance. Our findings corroborate and extend earlier propositions on LMS tracking data that found no consistent pattern of average time online in relation to course final grade [20]. Similarily Tempelaar and colleagues showed that LMS tracking data (such as simple clicking behavior) is only a weak proxy for student performance as multiple correlations of different performance indicators converged to value of about 0.2, indicating that no more than about 4% in performance variation can be explained by LMS track data [8]. Interestingly, while tracking data alone are not sufficient to draw conclusions about learner engagement, changes of student’s activity in virtual learning environments appears to be a valuable predictor [23], underscoring the importance of continuous measurement und collection of data about learners. More research is needed on the multivariate relationships between negatively correlating online assessment parameters and student academic performance. These negative indicators could be useful in determining how to support students through the provision of personalized feedback.

### Online assessments help students to judge their academic performance and level of knowledge

Enabling students to make accurate judgements about the quality of their work and their level of performance is one of the implicit aims of higher education [9]. Our study confirms earlier smaller-scale studies for traditional and web-based educational concepts that students’ judgements can be “calibrated” through continuous self-assessment and feedback, but overall remains a weak predictor of performance. Boud and colleagues showed that the overall students’ judgments converge with those of tutors, but with significant variation across achievement levels [9]. Similarily, Tousignant and DesMarchais reported a weak correlation of pre-exam self-assessment questionnaires with oral examinations (*r* ranging from 0.042 to 0.243) in a cohort of 70 students enrolled in a problem-based learning program of medicine [11].

### Online assessment parameters and their correlation to exam performance: role of gender

It has been asserted that the use of computers in higher education [13, 14] and learning outcomes in online courses [15–17] are increasingly gender neutral. However, it the area of computer-assisted assessments, research is not conclusive. Some researchers found that males do better in objective tests, including those based on MC-questions, which are often the mainstay of CAA [24, 25]. Other studies suggested that female students do worse than males because of anxiety or negative attitudes or anxiety towards computers [26–28]. In our study, we found no statistically significant differences between male and female students for predictive modelling of exam performances. Our results therefore back the assertions of Ory et al. [29] and Gunn et al. [13] that the use of CAA in higher education does not disadvantage different gender groups.

### What is the potential of online assessments in undergraduate medical education?

While this is an initial study conducted with a cohort of undergraduate medical students in pharmacology, it underscores that online assessments may provide a valuable tool for both students and educators in higher education to model and predict academic performance. At present, the mastery of a student in a particular subject is judged by summative assessments upon completion of a course. Typically, these aptitude tests provide the learner with a one-time feedback in the form of a final grade, at a time point in his studies, when all learning activities have already concluded [30]. In contrast, formative assessments provide both the learner and the teacher a continuous feedback during the learning and teaching process, respectively.

Thus, the research data reported in this study will be useful for further development of this and other online assessment platforms of pharmacology. This will likely result in improved formative feedback during the teaching and learning process and thus help to identify at-risk-students. However, further studies are needed to determine how students can be most efficently instructed based on the data from online formative assessments. This is particularly important in the context of pharmacology education, as pharmacotherapy-related topics were identified as areas of least confidence amongst first-year residents [2].

### Limitations of this study

While this study adds new insights in digital undergraduate medical education of pharmacology, there are limitations inherent to the methods applied in this study. Both assessment and self-evaluation questionnaires rely on self-report that may not be answered accurately or faithfully. Performance of students, who did not perform well in the online assessments, may have further underperformed in the summative examination due to demotivation and stress. Another limitation is that the use of a digital solution for collecting data may have led to a selection bias for students with higher affinity for digital technologies. Finally, our study cohort consisted of 393 undergraduate medical students enrolled in a basic pharmacology course at a single German medical school. The present study is therefore exploratory in nature and serves as a basis for future multicenter confirmatory studies with larger cohort sizes. Finally, further studies are needed to investigate if predictive models that incorporate the online assessment parameters identified in the present study will result in improved prediction of academic performance in undergraduate medical education of pharmacology.

## Conclusion

To our knowledge, this is the first prospective cohort study investigating online assessment parameters in undergraduate education of pharmacology and their correlation to summative exam performance. Our data suggest that already few and simple online assessments (e.g. score of the first attempt) can be helpful in identifying students that could benefit from remediation in a manner that is gender neutral. Moreover, our data suggest that formative feedback by online assessments help students to better judge their academic performance and level of knowledge. Further studies are needed to investigate if early implementation of online assessments during the teaching and learning phase as formative feedback source will result in improved outcome and knowledge retention in pharmacology.

## Supplementary information

**Additional file 1:** **Figure S1.** Structure and features of online assessment platform McPeer. An online assessment platform (designated “McPeer”) was developed for data acquisition of this study and made available via password-protected login to all students enrolled in the pharmacology course at TUM. A-C. Screenshots of website. A. Landing page (http://www.mcpeer.de). B. Individualized starting page after student login with overview of progress and testing performance of MC-question sets as shown by the percentage of correctly answered questions. C. An example of an MC-question.

**Additional file 2:** **Figure S2.** Screenshot of online questionnaire for self-evaluation of pharmacology knowledge. The online questionnaire was displayed after the first login to McPeer and 24 h before the final exam. Students were asked to rate their self-perceived competence on 27 topics that corresponded to the MC-question datasets on the learning analytics platform McPeer. A 5-point Likert-scale ranging from “confident” to “not confident” was employed. In addition, students could opt not to answer.

**Additional file 3:** **Figure S3.** Correlation of online assessment parameters with exam performance in pharmacology. Scatter plots depicting the correlation of exam performance vs. various learning analytics parameters (A-F). The coefficient of multiple correlations, R, was used as indicator of predictive modeling (*n* = 220).

**Additional file 4:** **Figure S4.** Subgroup analysis depicting the correlation of first and second administration of MC-questions with final exam score. Scatter plots illustrating the correlation of exam performance vs. results of first (A) and second (B) administration of MC-questions. The coefficient of multiple correlations, R, was used as indicator of predictive modeling (*n* = 46).

**Additional file 5:** **Figure S5.** Pre- and postintervention assessment of self-perceived pharmacology competency by male and female students. Online questionnaires were displayed at first login to McPeer (1. rating, preintervention) and 24 h before the final exam. A 5-point Likert-scale (1 = “insecure” to 5 = “secure”) was used. Differences between pairs of selfassessments (Likert Δ) as calculated by sign-tests before and after use of McPeer. Males = 47, Females = 103.

**Additional file 6:** **Table S1.** Topics and number of multiple-choice (MC) questions of the online assessment platform McPeer. ACE: Angiotensin-converting enzyme, NSAIDs: Nonsteroidal anti-inflammatory drugs.

**Additional file 7:** **Table S2.** Gender-specific analysis of various parameters and their predictive power of exam performance as bivariate correlation *r.*

## Data Availability

The data that support the findings of this study are openly available in Mendeley Data at 10.17632/wbp4tgtfmr.1

## Abbrevations

CAA: Computer-assisted assessment
LAK: International Conference on Learning Analytics and Knowledge
LMS: Learning management systems
MC: Multiple choice
MySQL: My Structured Query Language
PHP: Hypertext Preprocessor
SoLAR: Society for Learning Analytics Research
TUM: Technical University of Munich

## References

[1] Nikendei C (2009). Medical education in Germany. Med Teach.

[2] Ochsmann EB, Zier U, Drexler H, Schmid K (2011). Well prepared for work? Junior doctors’ self-assessment after medical education. BMC Med Educ.

[3] Ferguson R (2012). Learning analytics: drivers, developments and challenges. Int J Technol Enhanc Learn.

[4] Gutmann J, Kühbeck F, Berberat PO, Fischer MR, Engelhardt S, Sarikas A (2015). Use of learning media by undergraduate medical students in pharmacology: a prospective cohort study. PLoS One.

[5] Bienkowski M, Feng M, Means B (2012). Enhancing teaching and learning through educational data mining and learning analytics: an issue brief. US Dep Educ Office Educ Technol.

[6] Siemens G, Long P (2011). Penetrating the fog: analytics in learning and education. Educ Rev.

[7] Arbaugh JB (2014). System, scholar or students? Which most influences online MBA course effectiveness?. J Comput Assist Learn.

[8] Tempelaar DT, Rienties B, Giesbers B (2015). In search for the most informative data for feedback generation: learning analytics in a data-rich context. Comput Hum Behav.

[9] Boud D, Lawson R, Thompson DG (2013). Does student engagement in self-assessment calibrate their judgement over time?. Assess Eval High Educ.

[10] Boud D, Falchikov N, editors. Rethinking assessment in higher education: Learning for the longer term. Routledge: New York; 2007.

[11] Tousignant M, Desmarchais JE (2002). Accuracy of student self-assessment ability compared to their own performance in a problem-based learning medical program: a correlation study. Adv Health Sci Educ.

[12] Morris MG, Venkatesh V, Ackerman PL (2005). Gender and age differences in employee decisions about new technology: an extension to the theory of planned behavior. IEEE Trans Eng Manag.

[13] Gunn C, French S, McLeod H, McSporran M, Conole G (2002). Gender issues in computer-supported learning. Alt-J..

[14] Compton DM, Burkett WH, Burkett GG (2002). No sex difference in perceived competence of computer use among male and female college students in. Psychol Rep.

[15] Astleitner H, Steinberg R (2005). Are there gender differences in web-based learning? An integrated model and related effect sizes. AACE J.

[16] Lu J, Yu C-S, Liu C (2003). Learning style, learning patterns, and learning performance in a WebCT-based MIS course. Inf Manag.

[17] Yukselturk E, Bulut S (2007). Predictors for student success in an online course. J Educ Technol Soc.

[18] Statistical yearbook 2015, Federal Statistical Office of Germany (German: Statistisches Bundesamt, shortened Destatis); p. 693.

[19] Field A. Discovering statistics using IBM SPSS. Newbury Park: Sage Publications; 2009.

[20] Macfadyen LP, Dawson S (2010). Mining LMS data to develop an “early warning system” for educators: a proof of concept. Comput Educ.

[21] Mills EJ, Chan A-W, Wu P, Vail A, Guyatt GH, Altman DG (2009). Design, analysis, and presentation of crossover trials. Trials..

[22] Boud David (2013). Enhancing Learning Through Self-assessment.

[23] Wolff A, Zdrahal Z, Nikolov A, Pantucek M (2013). Improving retention: Predicting at-risk students by analysing clicking behaviour in a virtual learning environment.

[24] Birenbaum M, Feldman RA (1998). Relationships between learning patterns and attitudes towards two assessment formats. Educ Res.

[25] Hopkins S (2003). Assessment modes in first year macroeconomics: gender differences in performance. Econ Pap.

[26] Dambrot FH, Watkins-Malek MA, Silling SM, Marshall RS, Garver JA (1985). Correlates of sex differences in attitudes toward and involvement with computers. J Vocat Behav.

[27] Brosnan MJ (1998). The impact of psychological gender, gender-related perceptions, significant others, and the introducer of technology upon computer anxiety in students. J Educ Comput Res.

[28] Todman J (2000). Gender differences in computer anxiety among university entrants since 1992. Comput Educ.

[29] Ory JC, Bullock C, Burnaska K (1997). Gender similarity in the use of and attitudes about ALN in a university setting. J Asynchronous Learn Netw.

[30] Segers M, Dochy F, Cascallar E (2003). The era of assessment engineering: changing perspectives on teaching and learning and the role of new modes of assessment.

