# Supplementary material for: Correlation of online assessment parameters with summative exam performance in undergraduate medical education of pharmacology: a prospective cohort study
Source: BMC Med Educ. 2019 Nov 8;19:412. doi: 10.1186/s12909-019-1814-5 (PMC6842254; doi:10.1186/s12909-019-1814-5)
Supplement: Supplementary file 1 — Additional file 1: Figure S1. Structure and features of online assessment platform McPeer. An online assessment platform (designated “McPeer”) was developed for data acquisition of this study and made available via password-protected login to all students enrolled in the pharmacology course at TUM. A-C. Screenshots of website. A. Landing page (http://www.mcpeer.de). B. Individualized starting page after student login with overview of progress and testing performance of MC-question sets as shown by the percentage of correctly answered questions. C. An example of an MC-question. [file 12909_2019_1814_MOESM1_ESM.pdf]

Figure S1

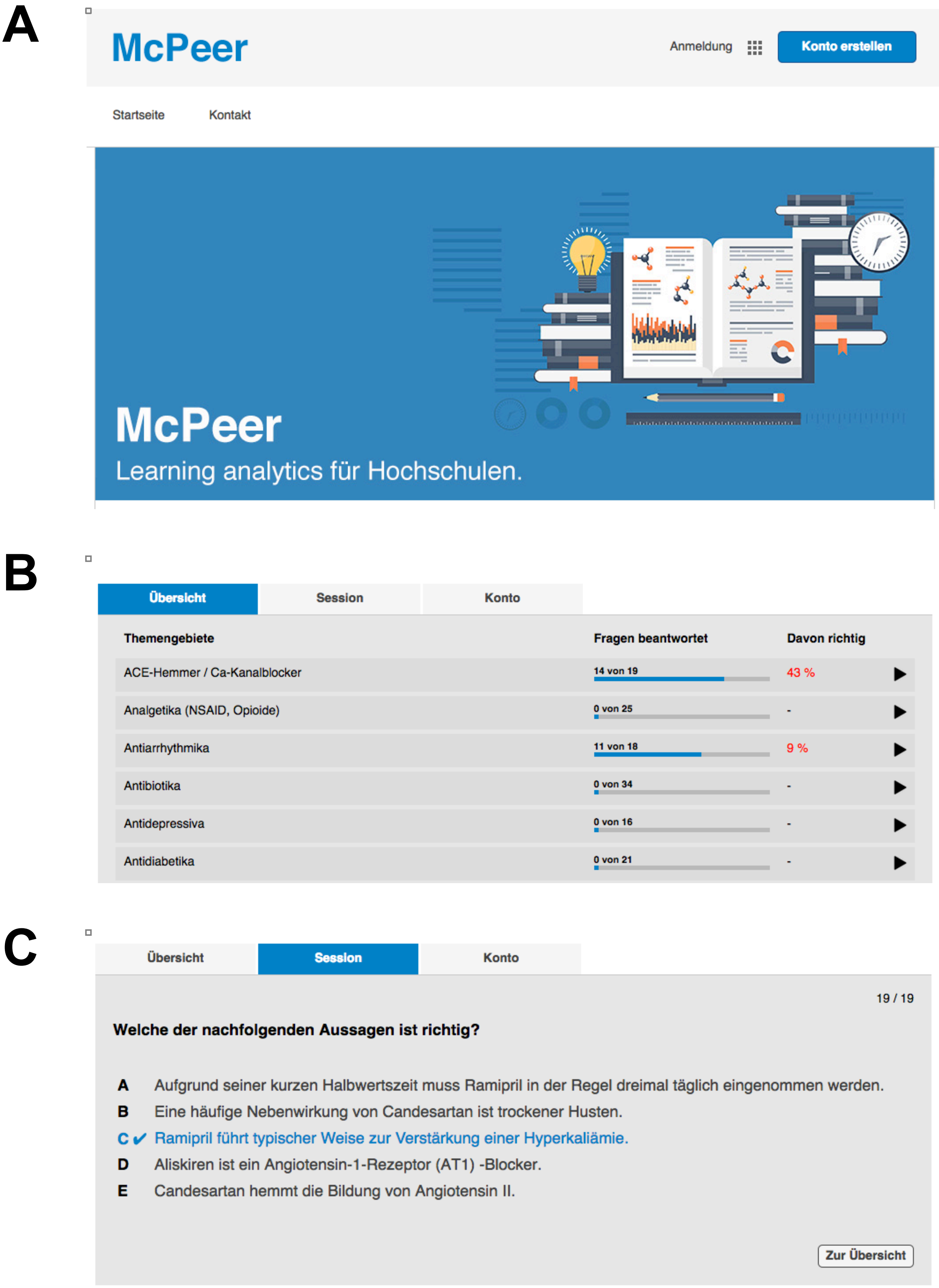
A
McPeer
Anmeldung
Konto erstellen
Startseite
Kontakt
McPeer
Learning analytics für Hochschulen.
B
Übersicht
Session
Konto
Themengebiete
Fragen beantwortet
Davon richtig
ACE-Hemmer / Ca-Kanalblocker
14 von 19
43 %
Analgetika (NSAID, Opioide)
0 von 25
-
Antiarrhythmika
11 von 18
9 %
Antibiotika
0 von 34
-
Antidepressiva
0 von 16
-
Antidiabetika
0 von 21
-
C
Übersicht
Session
Konto
19 / 19
Welche der nachfolgenden Aussagen ist richtig?
A Aufgrund seiner kurzen Halbwertszeit muss Ramipril in der Regel dreimal täglich eingenommen werden.
B Eine häufige Nebenwirkung von Candesartan ist trockener Husten.
C ✔ Ramipril führt typischer Weise zur Verstärkung einer Hyperkaliämie.
D Aliskiren ist ein Angiotensin-1-Rezeptor (AT1) -Blocker.
E Candesartan hemmt die Bildung von Angiotensin II.
Zur Übersicht
