# Supplementary material for: Correlation of online assessment parameters with summative exam performance in undergraduate medical education of pharmacology: a prospective cohort study
Source: BMC Med Educ. 2019 Nov 8;19:412. doi: 10.1186/s12909-019-1814-5 (PMC6842254; doi:10.1186/s12909-019-1814-5)
Supplement: Supplementary file 2 — Additional file 2: Figure S2. Screenshot of online questionnaire for self-evaluation of pharmacology knowledge. The online questionnaire was displayed after the first login to McPeer and 24 h before the final exam. Students were asked to rate their self-perceived competence on 27 topics that corresponded to the MC-question datasets on the learning analytics platform McPeer. A 5-point Likert-scale ranging from “confident” to “not confident” was employed. In addition, students could opt not to answer. [file 12909_2019_1814_MOESM2_ESM.pdf]

Figure S2

Themengebiete	Ich fühle mich in diesem Themengebiet...							Enthaltung
Antiparkinsonika	unsicher	○	○	○	○	○	sicher	○
Zytostatika und Tumorantikörper	unsicher	○	○	○	○	○	sicher	○
Glukokortiode/antiinflammatorische Biologika	unsicher	○	○	○	○	○	sicher	○
Analgetika (NSAID, Opioide)	unsicher	○	○	○	○	○	sicher	○
Neuroleptika	unsicher	○	○	○	○	○	sicher	○
GI-Trakt	unsicher	○	○	○	○	○	sicher	○
Kalziumstoffwechsel / Schilddrüse	unsicher	○	○	○	○	○	sicher	○
Antiemetika	unsicher	○	○	○	○	○	sicher	○
Antiarrhythmika	unsicher	○	○	○	○	○	sicher	○
Antiepileptika	unsicher	○	○	○	○	○	sicher	○
Diuretika	unsicher	○	○	○	○	○	sicher	○
Sympathikus	unsicher	○	○	○	○	○	sicher	○
Antituberkulotika / Antimalaria	unsicher	○	○	○	○	○	sicher	○
Pharmakokinetik / -genetik	unsicher	○	○	○	○	○	sicher	○
Antibiotika	unsicher	○	○	○	○	○	sicher	○
Narkose, Muskelrelaxantien	unsicher	○	○	○	○	○	sicher	○
Antidepressiva	unsicher	○	○	○	○	○	sicher	○
Pharmakodynamik	unsicher	○	○	○	○	○	sicher	○
Parasympathikus	unsicher	○	○	○	○	○	sicher	○
