# Supplementary figures and images for: Correlation of online assessment parameters with summative exam performance in undergraduate medical education of pharmacology: a prospective cohort study
Source: BMC Med Educ. 2019 Nov 8;19:412. doi: 10.1186/s12909-019-1814-5 (PMC6842254; doi:10.1186/s12909-019-1814-5)

**A**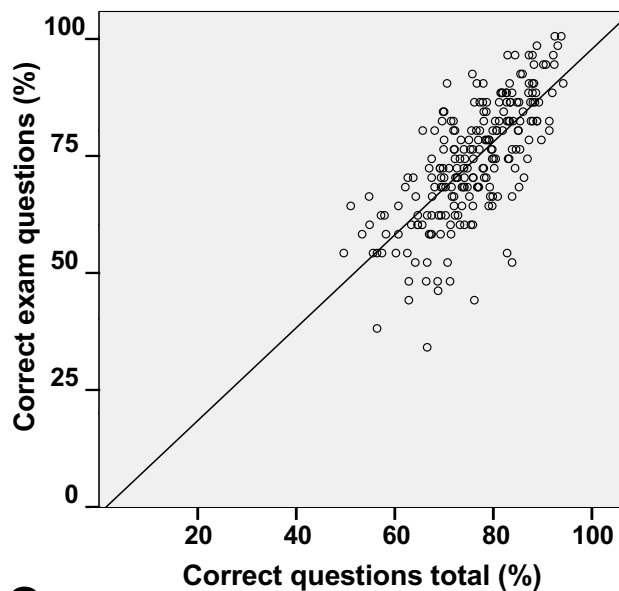**B**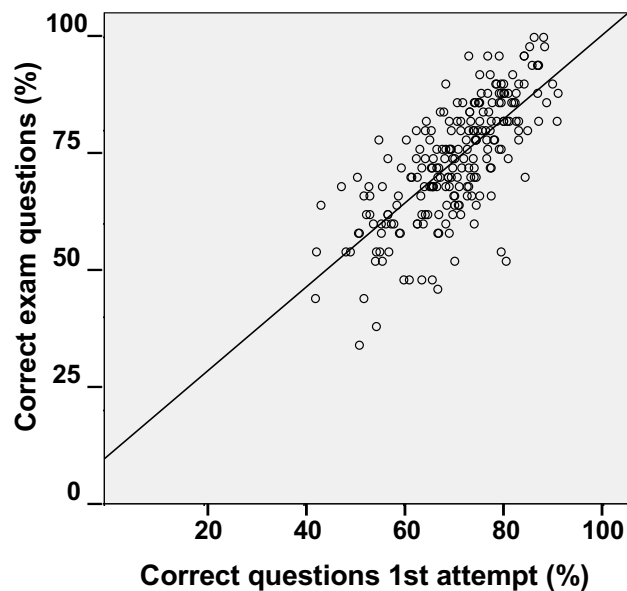**C**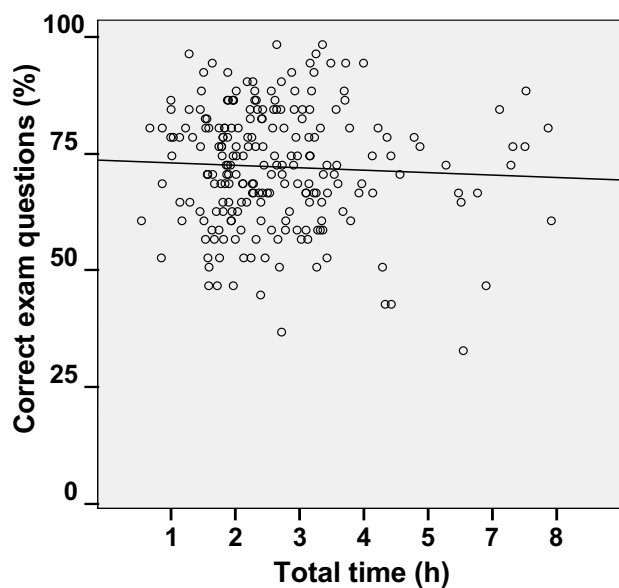**D**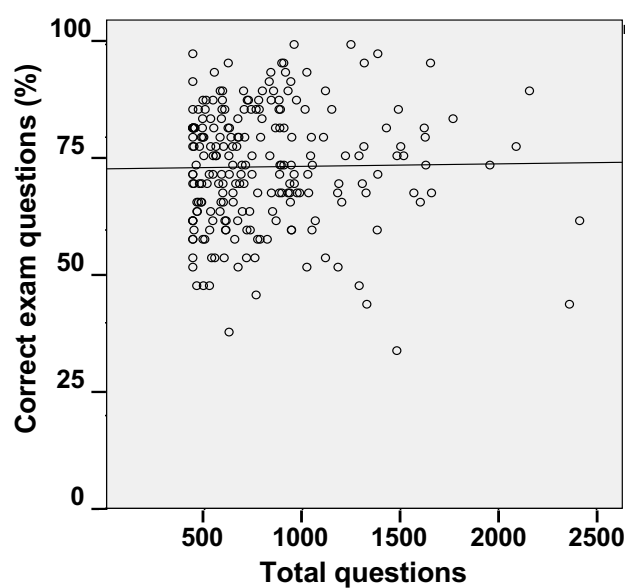**E**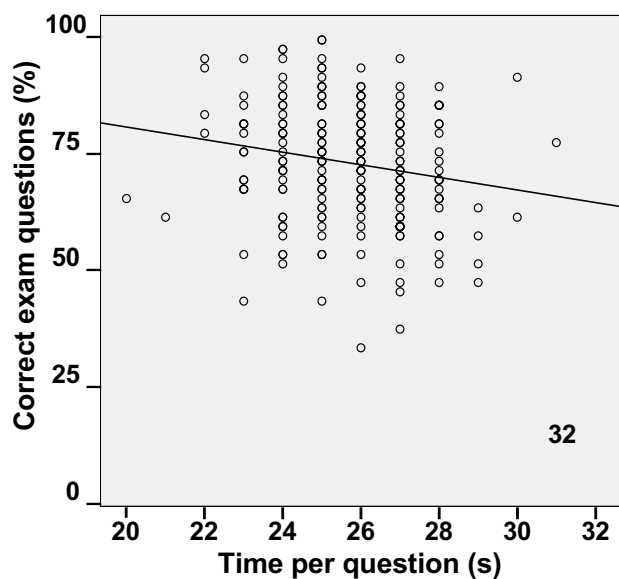**F**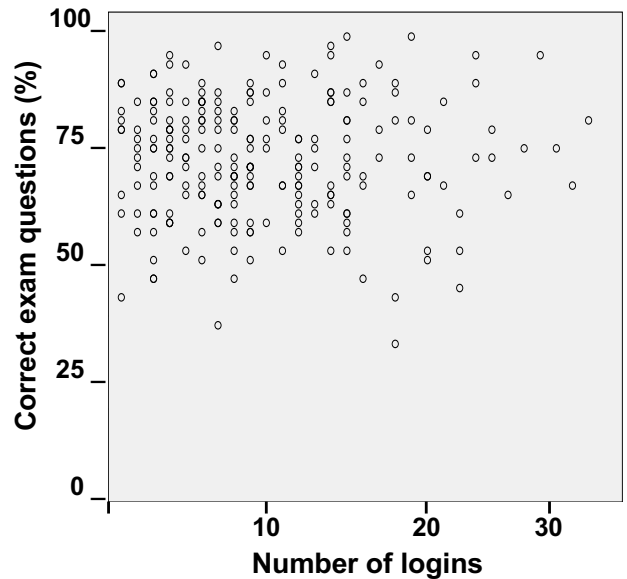

Supplement: Supplementary file 3 — Additional file 3: Figure S3. Correlation of online assessment parameters with exam performance in pharmacology. Scatter plots depicting the correlation of exam performance vs. various learning analytics parameters (A-F). The coefficient of multiple correlations, R, was used as indicator of predictive modeling (n = 220). [file 12909_2019_1814_MOESM3_ESM.pdf]

**A**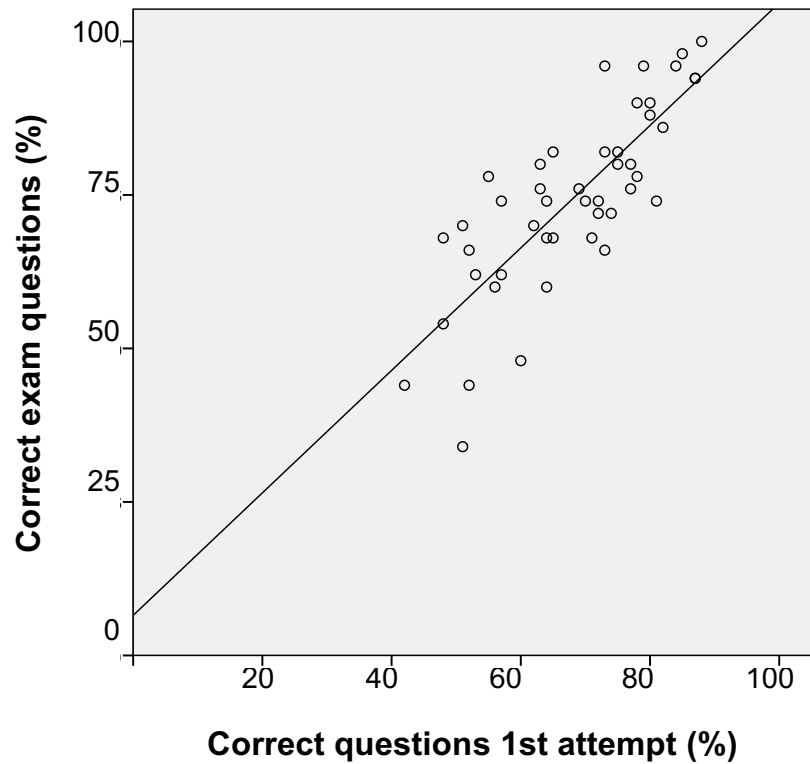**B**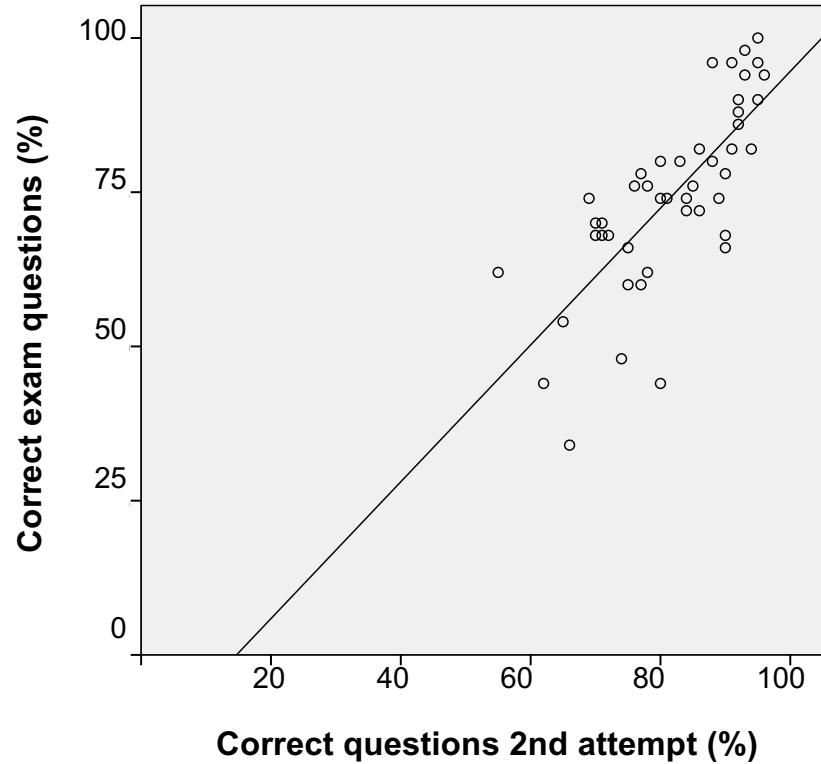

Supplement: Supplementary file 4 — Additional file 4: Figure S4. Subgroup analysis depicting the correlation of first and second administration of MC-questions with final exam score. Scatter plots illustrating the correlation of exam performance vs. results of first (A) and second (B) administration of MC-questions. The coefficient of multiple correlations, R, was used as indicator of predictive modeling (n = 46). [file 12909_2019_1814_MOESM4_ESM.pdf]

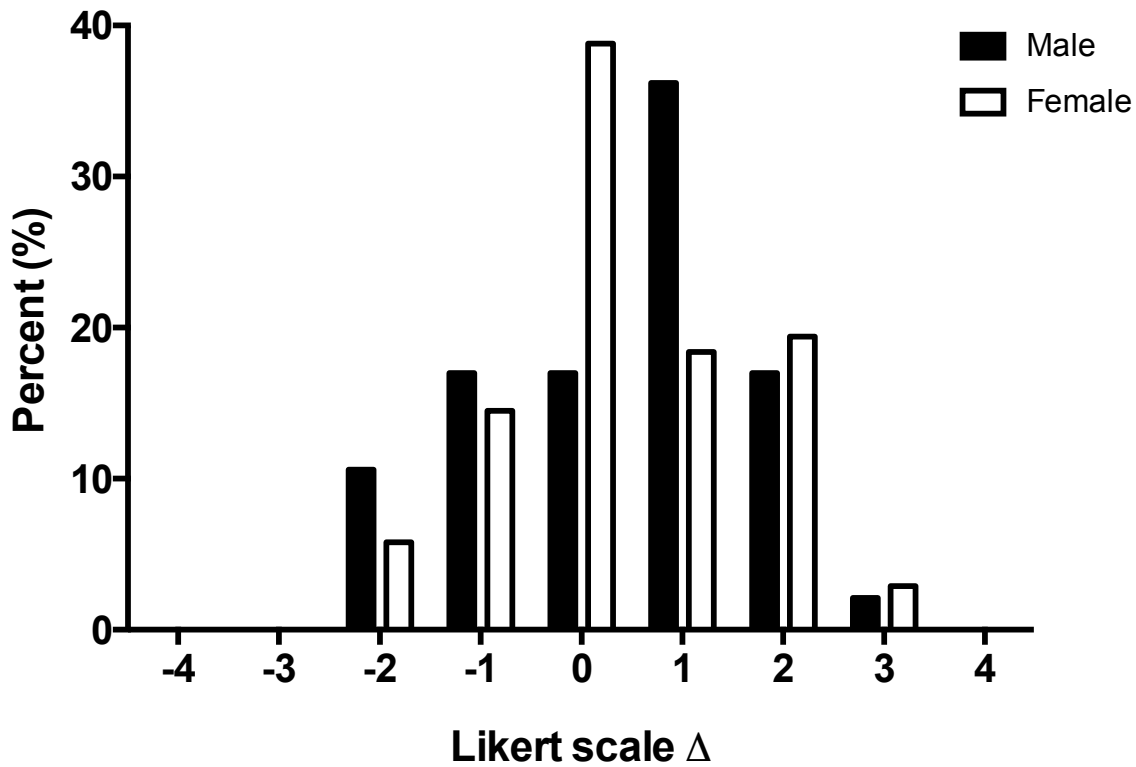

Supplement: Supplementary file 5 — Additional file 5: Figure S5. Pre- and postintervention assessment of self-perceived pharmacology competency by male and female students. Online questionnaires were displayed at first login to McPeer (1. rating, preintervention) and 24 h before the final exam. A 5-point Likert-scale (1 = “insecure” to 5 = “secure”) was used. Differences between pairs of selfassessments (Likert Δ) as calculated by sign-tests before and after use of McPeer. Males = 47, Females = 103. [file 12909_2019_1814_MOESM5_ESM.pdf]
