# Supplementary material for: Correlation of online assessment parameters with summative exam performance in undergraduate medical education of pharmacology: a prospective cohort study
Source: BMC Med Educ. 2019 Nov 8;19:412. doi: 10.1186/s12909-019-1814-5 (PMC6842254; doi:10.1186/s12909-019-1814-5)
Supplement: Supplementary file 6 — Additional file 6: Table S1. Topics and number of multiple-choice (MC) questions of the online assessment platform McPeer. ACE: Angiotensin-converting enzyme, NSAIDs: Nonsteroidal anti-inflammatory drugs. [file 12909_2019_1814_MOESM6_ESM.docx]

| **Topic** | **Number of MC-questions** |
| --- | --- |
|  |  |
| ACE inhibitors / Calcium channel blockers | 19 |
| Adrenergic agonists and antagonists | 26 |
| Analgesics (NSAIDs, opioids) | 25 |
| Anesthetics, muscle relaxants | 15 |
| Antiarrhythmic agents | 17 |
| Anticancer drugs | 27 |
| Antidepressants | 16 |
| Antidiabetic agents | 21 |
| Antidiarrheals, laxatives, gastric acid drugs | 14 |
| Antiemetics | 13 |
| Antiepileptic drugs | 11 |
| Antimalarial and antituberculosis agents | 17 |
| Antimicrobial drugs | 34 |
| Antiparkinson agents | 14 |
| Antiplatelet drugs, anticoagulants, thrombolytic drugs | 18 |
| Antipsychotics | 17 |
| Antiviral drugs, antifungal drugs | 16 |
| Calcium metabolism, thyroid hormones | 17 |
| Cholinergic agonists and antagonists | 10 |
| Corticosteroids, biologicals | 10 |
| Diuretics | 15 |
| Immunosuppressive drugs | 11 |
| Pharmacodynamics | 10 |
| Pharmacokinetics and -genetics | 14 |
| Sedatives | 16 |
| Steroid hormones | 11 |
| Toxins and antidotes | 6 |
|  |  |
| **Total** | **440** |

**Table S1: Topics and number of multiple-choice (MC) questions of the online assessment platform McPeer.** ACE: Angiotensin-converting enzyme, NSAIDs: Nonsteroidal anti-inflammatory drugs.
