# Supplementary material for: Correlation of online assessment parameters with summative exam performance in undergraduate medical education of pharmacology: a prospective cohort study
Source: BMC Med Educ. 2019 Nov 8;19:412. doi: 10.1186/s12909-019-1814-5 (PMC6842254; doi:10.1186/s12909-019-1814-5)
Supplement: Supplementary file 7 — Additional file 7: Table S2. Gender-specific analysis of various parameters and their predictive power of exam performance as bivariate correlation r. [file 12909_2019_1814_MOESM7_ESM.docx]

|  |
| --- |

**Table S2. Gender-specific analysis of various parameters and their predictive power of exam performance as bivariate correlation *r*.**

|  | **Male (n = 74)** | | | | **Female (n= 150)** | | | |
| --- | --- | --- | --- | --- | --- | --- | --- | --- |
| **Parameter** | **Mean  (± SD)** | **Median** | **bivariate correlation *r*** | **p-value** | **Mean  (± SD)** | **Median** | **bivariate correlation *r*** | **p-value** |
|  |  |  |  |  |  |  |  |  |
| **Number of logins** | 9.93  (± 8.08) | 7 [4;12] | 0.09 | 0.43 | 10.12  (± 6.44) | 9 [5;14] | - 0.04 | 0.65 |
| **Total questions** | 767.55  (± 303.26) | 689 [555;916] | - 0.02 | 0.85 | 836.64  (± 409.47) | 707 [252;975] | 0.03 | 0.75 |
| **Total score** | 74.87%  (± 9.21) | 74 [69;82] | 0.71 | < 0.05 | 75.74%  (± 9.19) | 76 [69;83] | 0.72 | < 0.05 |
| **Score first attempt** | 70.23%  (± 10.26) | 70 [64;79] | 0.77 | < 0.05 | 70.25%  (± 10.13) | 72 [64;77] | 0.71 | < 0.05 |
| **Total time** | 4.85 h  (± 1.47) | 4 [3;6] | - 0.14 | 0.25 | 5.06 h  (± 1.97) | 5 [4;6] | - 0.02 | 0.79 |
| **Time per question** | 25.80 s  (± 1.61) | 26 [25;27] | - 0.25 | < 0.05 | 25.67 s  (± 1.78) | 26 [24;27] | - 0.16 | 0.06 |
